# Interventions for recurrent embryo implantation failure: An umbrella review

**DOI:** 10.1002/ijgo.16066

**Published:** 2024-12-05

**Authors:** Abdulla Almohammadi, Fadi Choucair, Khalid S. Khan, Aurora Bueno‐Cavanillas, Naomi Cano‐Ibáñez

**Affiliations:** ^1^ Reproductive Medicine Unit Sidra Medicine Doha Qatar; ^2^ Department of Preventive Medicine and Public Health, Faculty of Medicine University of Granada Granada Spain; ^3^ Consortium for Biomedical Research in Epidemiology and Public Health (CIBERESP) Granada Spain; ^4^ Institute for Biosanitary Research IBs Granada Spain

**Keywords:** assisted reproductive technologies, live birth, meta‐analysis, pregnancy, randomized controlled trial, recurrent implantation failure

## Abstract

**Background:**

Recurrent implantation failure (RIF) has a multifactorial etiology. An umbrella review was undertaken to evaluate the multiple proposed interventions.

**Objectives:**

To summarize and assess the strength of evidence of interventions for RIF from published systematic reviews (SR) of randomized clinical trials (RCTs).

**Search Strategy:**

After prospective registration (PROSPERO CRD42023414255), a systematic search was conducted in the Cochrane Library, Scopus and Medline from inception until March 2024.

**Selection Criteria:**

SRs of RCTs, with or without meta‐analyses (MA), were included if they reported clinical pregnancy rates (CPR) or live birth rates (LBR).

**Data Collection and Analysis:**

The methodological quality of the included SRs was appraised independently in duplicate using the AMSTAR 2 tool. For each intervention, the MAs of RCTs with statistically significant improvements were counted as a percentage of the total assessing the strength of evidence using the GRADE system.

**Main Results:**

A total of 47 SRs were included: 32 reviews covered immunomodulatory interventions, 10 (68 RCTs) covered uterine and endometrial interventions, one covered antibiotics, and four (69 RCTs) addressed mixed approaches, including laboratory interventions. AMSTAR 2 appraised 41 (88%) SRs as critically low or low, and 6 (12%) as moderate or high in quality. The SRs often had a level of overlap of RCTs (median 33.3%), inconsistent definitions of RIF, and varied comparisons for the interventions. Considering the significant meta‐analytic evidence of high‐moderate GRADE strength: Granulocyte colony‐stimulating factor (G‐CSF) showed improvement in CPR in 9/13 (69.2%) MAs and LBR in 1/7 (14%); intralipid infusion showed improvement in CPR in 4/6 (57.14%) MAs and LBR in 3/4 (75%); peripheral blood mononuclear cells (PBMC) showed improvement in CPR in 4/8 (50%) MAs and LBR 3/5 (60%); platelet‐rich plasma (PRP) intervention showed improvement in CPR in 6/10 (60%) MAs and LBR in 1/5 (20%); human chorionic gonadotropins showed improvement in CPR in 3/3 (100%) MAs; growth hormone showed improvement in CPR in 1/1 (100%) MA; low molecular weight heparin showed improvement in CPR in 1/1 (100%) MAs and LBR in 1/2 (50%); hysteroscopy showed improvement in CPR in 1/2 (50%) MAs; and, intentional endometrial injury showed improvement in CPR in 3/4 (75%) MAs and LBR in 2/3 (66.66%).

**Conclusions:**

Evidence syntheses of RCTs evaluating interventions for RIF suggest a consistent direction, with high to moderate strength, indicating that immunomodulatory treatments, including G‐CSF, PBMC, PRP, intralipid infusion, and intentional endometrial injury are likely to be effective. However, this conclusion should be interpreted with caution due to the generally low methodological quality of the included studies and the clinical heterogeneity observed in most SRs.

## Synopsis

An umbrella review evaluating different interventions in treatment for recurrent implantation failure and their impact on clinical outcomes.

## 1 INTRODUCTION

Assisted reproductive techniques continue to evolve, many patients still experience serial in vitro fertilization (IVF) failures. Implantation is a complex process involving various factors related to the embryo, endometrium, and the immune system.^1^, ^2^ Recurrent implantation failure (RIF) presents a challenging condition characterized by the inability to achieve pregnancy, determined by persistently negative human chorionic gonadotropin levels, even after undergoing multiple embryo transfers (ETs).^3^, ^4^, ^5^ While definitions of RIF agree on the recurrent nature of the condition, there is a lack of consensus on the specific number of failed attempts.^2^, ^6^ The pathogenesis of RIF is multifaceted,^2^ and numerous interventions have been proposed for its management.^7^, ^8^

The European Society for Human Reproduction and Embryology (ESHRE) developed good practice recommendations in early 2023 for RIF management.^9^ These recommendations were an amalgam of expert opinions and a literature review (PubMed 1950–August 10, 2022), prioritizing randomized clinical trials (RCTs) but also including relevant observational studies without evidence grading.^10^ Several network meta‐analyses have ranked interventions for their effectiveness in different orders.^11^, ^12^, ^13^, ^14^, ^15^ Thus, there are varying recommendations. An umbrella review with evidence grading of systematic reviews (SR) and meta‐analyses (MA) could aid in interpreting these findings and provide clinicians with a more thorough and transparent evidence‐based approach.

The objective of this umbrella review was to systematically review and assess the strength of evidence pertaining to interventions for the management of RIF within SRs of RCTs reporting clinical pregnancy rates (CPR) or live birth rates (LBR) as outcomes.

## 2 METHODS

We conducted this overview of SRs after prospective registration (PROSPERO no. CRD42023414255) and reported it in accordance with the PRISMA (Preferred Reporting Items for Meta‐analyses guidelines)^16^ (Table S1).

### 2.1 Search strategy and selection criteria

A comprehensive search strategy without language restriction was applied in electronic databases (Medline, Scopus, Cochrane Library) from inception until March 25, 2024. We used a combination of keywords and free text terms including “Recurrent implantation failure,” “implantation failure,” “meta‐analyses,” “meta‐analysis” and “randomized controlled trials.” The search terms are presented in Appendix S1. All citations found were exported to Endnote software (version X9), where duplicates were removed. Two reviewers (AI and FC) carried out the searches independently and screened all abstracts and titles using Rayyan software. We included SRs comprising RCTs assessing interventions (stand‐alone interventions compared to a placebo or no treatment) for RIF. The SR with MA encompassed interventions for RIF treatment, defined a priori within categories in the latest ESHRE recommendations (2023) (9), regardless of the specific definition of RIF used. Four nodes were identified to classify these interventions: (i) interventions based on diagnostic findings (include antibiotic treatment); (ii) immunomodulatory interventions (include intravenous immunoglobulin (IVIG)); peripheral blood mononuclear cells (PBMC) infusion; subcutaneous or intrauterine granulocyte colony‐stimulating factor (G‐CSF) administration; intrauterine autologous platelet‐rich plasma (PRP) infusion; intravenous intralipid; intrauterine human chorionic gonadotropins (hCG) injection; low molecular weight heparin (LMWH); (iii) uterine and endometrial interventions (include: intentional endometrial injury; hysteroscopy; GnRH agonist and aromatase inhibitor pretreatment; sildenafil); and (iv) laboratory interventions (include: preimplantation genetic testing for aneuploidy (PGT‐A)). The clinical outcomes of interest were clinical pregnancy rates (CPR) or live birth rates (LBR) reported as either primary or secondary outcomes. The exclusion criteria were reviews and studies which did not report these outcomes, and their designs were other than SR or MA, that is, narrative reviews and reviews of non‐RCT evidence. Any disagreement regarding the inclusion of the citations was resolved by obtaining the opinion of a third researcher (NC‐I).

### 2.2 Data extraction, methodological quality assessment and evidence grading

The characteristics of selected studies, methodological quality assessment and evidence grading was extracted by AI and checked for accuracy by FC after reading the full text. Extracted data included citation details (author and year), type of intervention, comparator, the number of RCTs included, number of participants, the ratings of their quality, and pooled effect sizes with their 95% confidence intervals (CI) for the outcome: CPR or LBR. The pooled effect estimates, and heterogeneity of estimates were those reported within each included MA. The methodological quality of the included SRs was independently assessed by two reviewers (AI and FC) using the 16‐item questionnaire, that is, a measurement tool for assessment of multiple systematic reviews (AMSTAR‐2).^17^ Disagreements were resolved via consultation with a third reviewer (NC‐I). According to the guidelines, the reviewers assigned one of four global quality ratings (i.e., high, moderate, low or critically low) after the consideration of 16 potential critical and noncritical weaknesses. High and moderate ratings reflected the presence of one or less or one noncritical weakness, respectively. Low and critically low ratings indicated one or more than one critical weakness, respectively.

Using Grading of Recommendations Assessment, Development and Evaluation (GRADE) methodology,^10^ the strength of evidence was categorized as high, moderate, low, or critically low, considering five domains: risk of bias of included studies, indirectness of evidence, inconsistency of results (heterogeneity), imprecision of results and possibility of publication bias.^18^ The risk of bias was assessed based on the quality of the included RCTs, with grading categorized as “not serious,” “with serious limitations,” or “with very serious limitations,” depending on the assessment tool used by the included MA. Indirectness was evaluated through the population, intervention, comparator, outcome (PICO) framework. If the MA clearly defined these elements, the domain was considered “not serious.” Inconsistency was assessed using the I^2^ score, with a score above 50% classified as a “serious limitation” and a score exceeding 80% considered a “very serious limitation.” Imprecision was evaluated based on point estimates, CIs, and *P* values, with thresholds set around 0.75 and 1.25.^19^ A point estimate below 1 with an upper CI limit exceeding 1.25, or a point estimate of 1 or higher with a lower CI limit falling below 0.75, was marked as a “serious limitation.” Finally, for assessing publication bias, data from the funnel plots and Egger tests reported in the included MAs were utilized.^18^

### 2.3 Data synthesis

The extracted data in each SR were structured and the findings were tabulated, including the overall number of RCTs and participants. Significant summary results were grouped by intervention and outcomes to present an overview of direction and magnitude of effect. The corrected covered area (CCA) was calculated to quantify the degree of overlap between SRs. Analysis of overlap was performed by groups of MAs determined by: (a) The type of evaluated interventions, and (b) the reported pre‐specified outcomes of the study: CPR and live birth rates. CCA is a percentage, calculated as (N − r)/(rc − r), where *N* is the number of publications included in the evidence synthesis, *r* is the number of rows and *c* is the number of columns. CCA below 5% was considered a slight overlap, a CCA >5 and ≤10% a moderate overlap, a CCA >10 and ≤15% a high overlap, and a CCA >15% as a very high overlap.^20^ The results with statistically significant improvements for each intervention were counted as a percentage of the total taking into account the evidence strength assessed by GRADE. With many interventions, vote‐counting conducted within broad subgroups stratified by evidence strength assessment is known to minimize bias by incorporating quality. It has previously been used to synthesize whether evidence showed improvement or no change.^21^, ^22^, ^23^

## 3 RESULTS

**FIGURE 1:**
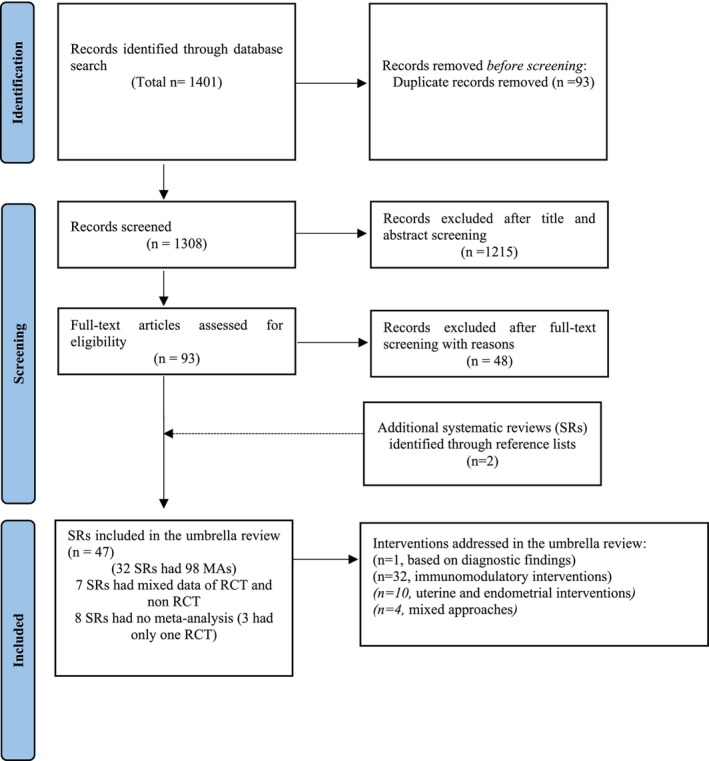
Flow chart of the systematic reviews included in the umbrella review of interventions for the treatment of recurrent embryo implantation failure.

Of the total of 1401 records were initially identified, 47 SRs met the eligibility criteria including a total of 375 RCTs. Out of 47, eight SRs did not have MAs; of these, three SRs had only one RCT each. Seven SRs had unextractable MA data for RCTs only. Out of the included 32 SRs with MA, six SRs had multiple MAs.^7^, ^11^, ^12^, ^13^, ^14^, ^15^ There were 98 MAs of RCTs. Figure 1 presents the PRISMA^16^ flow diagram of the selection process. A list of studies excluded from the overview with the main reason for their exclusion are summarized in Table S2.

### 3.1 Characteristics of the included systematic reviews

The main characteristics of the selected SRs are described in Table S3. All included SRs were published between 2012 and 2023, and their searches for RCTs were conducted between 2012 and 2022. Only one of the SRs was a Cochrane Review.^24^ Total number of MAs for CPR were 59 and that for LBR were 39. Of the total SRs, 32 (237 RCTs) covered immunomodulatory interventions, 10 (68 RCTs) covered uterine and endometrial interventions, one (1 RCT) covered antibiotics, and four (69 RCTs) covered mixed approaches.

### 3.2 Methodological quality assessment and overlap of evidence

**FIGURE 2:**
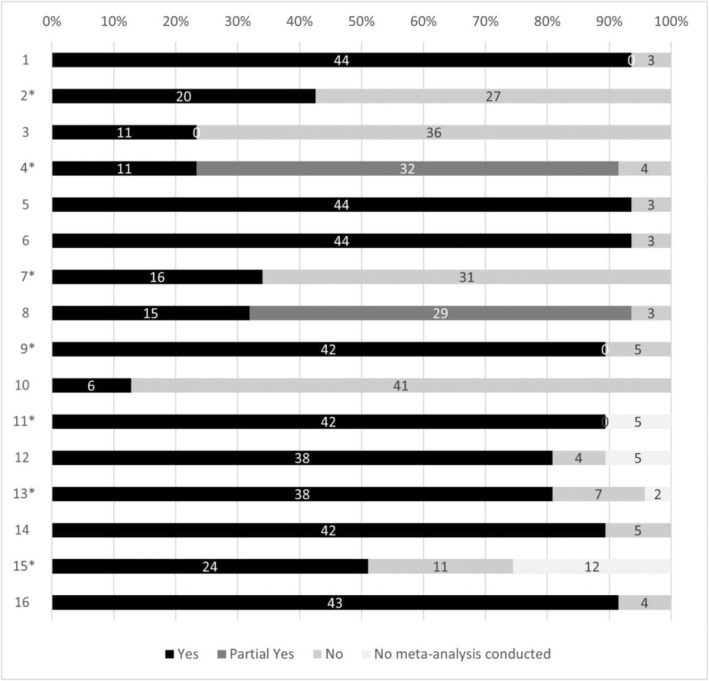
Methodological quality of 47 Systematic reviews included in the umbrella review of interventions for the treatment of recurrent embryo implantation failure. Quality assessment according to the 16 items of AMSTAR 2 (see Section 2 for details). Item 1: Research questions and inclusion criteria include PICO components; Item 2: Previous protocol review; Item 3: Explaining decision about the study designs to include in the review; Item 4: Adequate literature search; Item 5: Study selection performed in duplicate; Item 6: Data extraction performed in duplicate; Item 7: Excluded studies justification; Item 8: Describing included studies with sufficient detail; Item 9: Bias risk of individual studies included; Item 10: Reporting the sources of funding for the studies included in the review; Item 11: Appropriate meta‐analysis methods; Item 12: Assessing the potential impact of bias risk on results; Item 13: Consideration of the bias risk in the interpretation of the review results; Item 14: Satisfactory explanation and discussing any observed heterogeneity in the review results; Item 15: Assessment of the presence and probable impact of publication bias; and Item 16: Potential sources of conflict including any funding received. *Critical items: 2, 4, 7, 9, 11, 13 and 15; Non‐critical items: 1, 3, 5, 6, 8, 10, 12, 14 and 16.

Using AMSTAR 2 tool (Table S4), three SRs (6%) were rated as high methodological quality,^25^, ^26^, ^27^ three were rated as moderate methodological quality (6%),^28^, ^29^, ^30^ 19 (41%) were evaluated as low quality,^7^, ^14^, ^15^, ^24^, ^31^, ^32^, ^33^, ^34^, ^35^, ^36^, ^37^, ^38^, ^39^, ^40^, ^41^, ^42^, ^43^, ^44^, ^45^ and 22 (47%) were critically low quality.^11^, ^12^, ^13^, ^38^, ^46^, ^47^, ^48^, ^49^, ^50^, ^51^, ^52^, ^53^, ^54^, ^55^, ^56^, ^57^, ^58^, ^59^, ^60^, ^61^, ^62^, ^63^ The high‐quality reviews were conducted for immunomodulatory interventions G‐CSF^26^; growth hormone (GH); PRP.^25^ Regarding the SRs with moderate quality, one pertained to PBMC,^29^ another focused on G‐CSF,^28^ and one SR was related to endometrial scratch.^30^ Among the AMSTAR‐2 criteria (Figure 2), the most frequent critical weakness identified were the lack of a previous protocol review (27 SRs), the exclusion studies without robust justification (31 SRs); and an inappropriate investigation of publication bias (11 SRs). The AMSTAR‐2 assessment of methodological quality revealed that the majority of the included SRs (42 out of 47) described the risk of bias in the incorporated studies. A degree of overlap was found between SRs, with a median CCA of 33.3% (IQR 45.15%, min 0%–max 100%) (Table 1). Tables 2 and 3 show the strength of the evidence between proposed interventions and each selected clinical outcome.

**TABLE 1:** Overlapping between meta‐analyses included in the umbrella review of interventions for the treatment of recurrent embryo implantation failure.

Overlapping	*N*	CCA	Classification
Immunomodulatory interventions			
Reviews of RCTs for intralipid effects on CPR in RIF patients	6	52%	Very high
Reviews of RCTs for intralipid effects on LBR in RIF patients	6	52%	Very high
Reviews of RCTs for PRP effects on CPR in RIF patients	6	38.30%	Very high
Reviews of RCTs for PRP effects on LBR in RIF patients	6	30%	Very high
Reviews of RCTs for PBMC effects on CPR in RIF patients	5	33.3%	Very high
Reviews of RCTs for PBMC effects on LBR in RIF patients	5	0%	Low
Reviews of RCTs for G‐CSF effects on CPR in RIF patients	8	33.3%	Very high
Reviews of RCTs for G‐CSF effects on LBR in RIF patients	8	0%	Low
Reviews of RCTs for IVIG effects on CPR in RIF patients	2	100%	Very high
Reviews of RCTs for IVIG effects on LBR in RIF patients	2	0%	Low
Uterine and endometrial interventions			
Reviews of RCTs for hysteroscopy effects on CPR in RIF patients	2	0%	Low
Reviews of RCTs for hysteroscopy effects on LBR in RIF patients	2	33.3%	Very high
Laboratory interventions			
Reviews of RCTs for PGT‐A effect on CPR in RIF patients	2	100%	Very high
Reviews of RCTs for PGT‐A effect on LBR in RIF patients	2	100%	Very high

*Note*: (CCA = (*N* − *r*)/(*rc* − *r*)).

Abbreviations: CCA, corrected covered area; CPR, clinical pregnancy rate; G‐CSF, granulocyte colony‐stimulating factor; IVIG, intravenous immunoglobulins; LBR, live birth rate; *N*, number of reviews included in the overlapping assessment; PGT‐A, preimplantation genetic testing for aneuploidy; PBMC, peripheral blood mononuclear cells; PRP, platelet‐rich plasma; RCTs, randomized clinical trials; RIF, recurrent implantation failure.

**TABLE 2:** Certainty of evidence for various interventions reported by meta‐analyses on the outcome clinical pregnancy rate in the umbrella review of interventions for the treatment of recurrent embryo implantation failure.

Study ID	Intervention	Comparison (No. of RCTs)	Total no. RCTs	No. of patients with CP/total no.	Summary estimate (95% CI) reported for RCT data in the included reviews	GRADE assessment	Reasons for downgrading	AMSTAR‐2 rating
Immunomodulatory interventions								
G‐CSF								
Kamath et al. 2020^24^	G‐CSF mixed routes	Placebo (2) or no intervention (4) or mixed (1)	7	150/643	RR, 2.11 (1.56–2.85)	Low	Serious ROB, strongly suspected publication bias	Low
Kamath et al. 2017^33^	G‐CSF sub‐cutaneous	Placebo (2)	2	39/139	RR, 2.51 (1.36–4.63)	High	NA	Low
Zhang et al. 2018^42^	G‐CSF mixed routes	Placebo (3) or no intervention (3) or mixed (2)	8	NR/NR	RR, 2.07 (1.64–2.61)	Moderate	Serious ROB	Low
Jiang et al. 2020^52^	G‐CSF mixed routes	Placebo (4) or no intervention (5) or mixed (2)	11	NR/1035	RR, 1.91 (1.56–2.34)	Moderate	Serious ROB	Critically Low
Wang et al. 2021^13^	G‐CSF mixed routes	Placebo (3) or no intervention (2) or mixed (1)	6	NR/NR	OR, 2.03 (1.35–3.05)^a^	Low	Serious inconsistency, serious indirectness	Critically Low
Busnelli et al. 2021^7^	G‐CSF sub‐cutaneous	Placebo (2) or no intervention (2)	4	99/333	RR, 2.29 (1.58–3.31)	Moderate	Serious ROB	Low
Busnelli et al.2021^7^	G‐CSF intrauterine	Placebo (1) or mixed (1)	2	65/257	RR, 1.53 (1.00–2.33)	Moderate	Serious ROB	Low
Hou et al. 2021^28^	G‐CSF mixed routes	Placebo (4) or no intervention (4) or mixed (1)	9	NR/986	RR, 1.55 (1.3–1.85)	High	NA	Moderate
Liu et al. 2022^12^	G‐CSF intrauterine	Placebo (3) or no intervention (1)	4	NR/411	OR, 3.81 (2.53–5.72)^a^	Moderate	Serious inconsistency	Critically Low
Jin et al.2022^11^	G‐CSF mixed routes	Placebo (3) or no intervention (2)	5	NR/547	OR, 1.93 (1.37–2.72)^a^	Low	Serious inconsistency, very serious indirectness	Critically Low
Liu et al. 2022^12^	G‐CSF sub‐cutaneous	Placebo (2)	2	NR/164	OR, 6.69 (3.45–12.97)^a^	Moderate	Serious imprecision	Critically Low
Lu et al. 2023^26^	G‐CSF mixed routes	Placebo (9) or no intervention (5)	14	419/1387	RR, 1.93 (1.63–2.29)	Moderate	Serious ROB	High
Kong et al. 2023^14^	G‐CSF	Placebo (4) or no intervention (2)	6	NR/923	OR, 0.63 (0.44–0.89)^a^	Low	Serious inconsistency, serious indirectness	Low
He et al. 2023^15^	G‐CSF	Placebo (11) or no intervention (3)	14	NR/1291	OR, 1.94 (1.49–2.55)^a^	Low	Serious ROB, serious inconsistency	Low
GH								
Vera‐Montoya et al. 2023^27^	Growth hormone	No intervention (2)	2	37/112	OR, 4.97 (2.05–12.05)	High	NA	High
IVIG								
Achilli et al. 2018^46^	IVIG	Placebo (2)	2	22/90	OR, 1.09 (0.32–3.68)	Moderate	Serious imprecision	Critically low
Wang et al. 2021^13^	IVIG	Placebo (2)	2	NR/100	OR, 1.07 (0.38–2.98)^a^	Low	Serious inconsistency, serious indirectness, serious imprecision	Critically low
hCG								
Xie et al. 2019^63^	hCG	No intervention (3)	3	NR/417	RR, 1.33 (1.03–1.73)	High	NA	Critically Low
Jin et al. 2022^11^	hCG	No intervention (2)	2	NR/255	OR, 1.8 (1.18–2.72)^a^	Moderate	Serious inconsistency	Critically Low
Kong et al. 2023^14^	hCG	Placebo (3) or no intervention (4)	4	NR/668	OR, 0.62 (0.45–0.87)^a^	Low	Serious inconsistency, Serious indirectness	Low
He et al. 2023^15^	hCG	No intervention (4)	4	NR/858	OR, 1.61 (1.15–2.29)^a^	Moderate	Serious inconsistency	Low
Intralipid infusion								
Zhou et al. 2020^43^	Intralipid infusion	Placebo (2) or no intervention (2)	4	153/544	RR, 1.74 (1.27–2.4)	Moderate	Serious ROB	Low
Wang et al. 2021^13^	Intralipid infusion	Placebo (1) or no intervention (1)	2	NR/244	OR, 1.98 (1.02–3.64)^a^	Low	Serious inconsistency, serious indirectness	Critically Low
Han et al. 2021^32^	Intralipid infusion	Placebo (3) or no intervention (2)	5	291/840	RR, 4.48 (1.23–1.79)	High	NA	Low
Rimmer et al. 2021^45^	Intralipid infusion	Placebo (3) or no intervention (2)	5	50/203	RR, 1.55 (1.16–2.06)	High	NA	Critically low
Kumar et al. 2021^53^	Intralipid infusion	Placebo (2) or no intervention (3) or mixed (1)	6	213/560	OR, 1.51 (1.06–2.13)	Moderate	Very serious ROB	Critically Low
He et al. 2023^15^	Intralipid infusion	No intervention (2)	2	NR/345	OR, 2.18 (1.2–3.94)^a^	Low	Serious ROB, serious inconsistency	Low
LMWH								
Liu et al. 2022^12^	LMWH	No intervention (2)	2	NR/357	OR, 2.94 (2.01–4.31)^a^	Moderate	Serious ROB	Critically Low
Busnelli et al. 2021^7^	LMWH	No intervention (2)	2	49/218	RR, 1.39 (0.87–2.23)	Moderate	Very serious ROB	Low
He et al. 2023^15^	LMWH	No intervention (1)	4	NR/634	OR, 1.2 (0.83–1.72)^a^	Moderate	Serious inconsistency	Low
PBMC								
Wu et al. 2019^62^	PBMC	No intervention (3)	3	105/316	OR, 2.45 (1.53–3.91)	High	NA	Critically Low
Maleki‐Hajiagha et al. 2019^57^	PBMC	No intervention (2)	2	76/225	RR, 2.32 (1.57–3.44)	Low	Very serious ROB	Critically Low
Pourmoghadam et al. 2020^29^	PBMC	No intervention (2)	2	76/225	OR, 3.57 (1.99–6.4)	High	NA	Moderate
Wanget al. 2021^13^	PBMC	No intervention (5)	5	NR/639	OR, 2.63 (1.71–4.06)^a^	Low	Serious inconsistency, serious indirectness	Critically Low
Busnelli et al. 2021^7^	PBMC	No intervention (3)	3	114/363	RR, 2.18 (1.58–3)	Low	Very serious ROB	Low
Liu et al. 2022^12^	PBMC	No intervention (4)	4	NR/602	OR, 6.08 (4.37–8.45)^a^	Low	Very serious ROB	Critically Low
Jin et al. 2022^11^	PBMC	No intervention (3)	3	NR/366	OR, 2.79 (1.75–4.45)^a^	Moderate	Serious inconsistency	Critically Low
Kong et al. 2023^14^	PBMC	No intervention (4)	4	NR/450	OR, 0.42 (0.26–0.69)^a^	Low	Serious ROB, Serious inconsistency, Serious indirectness	Low
He et al. 2023^15^	PBMC	No intervention (6)	6	NR/605	OR 3.17 (2.08–4.87)^a^	Moderate	Serious inconsistency	Low
PRP								
Busnelli et al. 2021^7^	PRP	No intervention (2)	2	69/195	RR, 2.45 (1.55–3.86)	Low	Very serious ROB	Low
Wang et al. 2021^13^	PRP	Placebo (3)	3	NR/245	OR, 2.55 (1.36–4.79)^a^	Low	Serious inconsistency, serious indirectness	Critically Low
Liu et al. 2022^55^	PRP	Placebo (3) or no intervention (2)	5	222/718	OR, 3.66 (2.58–5.19)	High	Serious ROS	Critically Low
Liu et al. 2022^12^	PRP	Placebo (1) or no intervention (1)	2	NR/195	OR, 6.73 (3.68–12.31)^a^	High	Serious imprecision	Critically Low
Li et al. 2022^34^	PRP	Placebo (2) or no intervention (4)	6	383/1056	OR, 2.98 (2.29–3.88)	High	NA	Low
Hu et al. 2023^51^	PRP	Placebo (2) or no intervention (2)	4	238/678	RR, 2.46 (1.93–3.12)	Low	Very serious ROB	Critically Low
Jin et al. 2022^11^	PRP	Placebo (2) or no intervention (2)	4	NR/755	OR, 3.78 (2.72–5.25)^a^	Moderate	Serious inconsistency	Critically Low
Maged et al. 2023^35^	PRP	Placebo (1) or no intervention (5)	6	148/501	OR, 1.95 (1.1–3.46)	Moderate	Serious ROB	Low
Anitua et al. 2023^25^	PRP	Placebo (3) or no intervention (4)	7	284/893	RR, 2.18 (1.76–2.7)	High	NA	High
Kong et al. 2023^14^	PRP	Placebo (2) or no intervention (5)	7	NR/958	OR, 0.41 (0.26–0.66)^a^	Low	Serious ROB, Serious inconsistency, Serious indirectness	Low
He et al. 2023^15^	PRP	Placebo (3) or no intervention (2)	5	NR/758	OR 3.22 (2.13–4.78)^a^	Low	Serious ROB, serious inconsistency	Low
Uterine and endometrial interventions								
Hysteroscopy								
Mao et al. 2019^59^	Hysteroscopy	No intervention (3)	3	NR/1581	OR, 1.5 (1.06–2.12)	Moderate	Serious inconsistency	Critically Low
He et al. 2023^15^	Hysteroscopy	No intervention (4)	4	NR/1901	OR, 1.76 (1.34–2.39)^a^	Low	Very serious ROB, serious inconsistency	Low
Intentional endometrial injury								
Potdar et al. 2012^36^	Intentional endometrial injury	No intervention (4)	4	275/983	RR, 1.71 (1.4–2.09)	High	NA	Low
Vitagliano et al. 2018^30^	Intentional endometrial injury	Placebo (1) or no intervention (7)	8	206/817	RR, 1.57 (1.22–2.03)	High	NA	Moderate
Sar‐Shalom Nahshon et al. 2019^39^	Intentional endometrial injury	No intervention (5)	5	136/522	RR, 1.53 (0.93–2.51)	Low	Serious ROB, serious inconsistency	Low
Busnelli et al. 2021^7^	Intentional endometrial injury	No intervention (3)	3	100/286	RR, 1.43 (0.79–2.61)	Moderate	Serious inconsistency	Low
Jin et al. 2022^11^	Intentional endometrial injury	No intervention (6)	6	NR/1006	OR, 1.75 (1.29–2.36)^a^	Moderate	Serious inconsistency	Critically Low
Maged et al. 2023^44^	Intentional endometrial injury	No intervention (9)	9	325/1032	OR, 1.24 (0.77–2.02)	Low	Very serious ROB, serious inconsistency	Low
He et al. 2023^15^	Intentional endometrial injury	No intervention (6)	6	NR/718	OR, 1.83 (1.28–2.64)^a^	Low	Very serious ROB, serious inconsistency	Low
Laboratory interventions								
PGT‐A								
Busnelli et al. 2021^7^	PGT‐A	No intervention	2	85/230	RR 1.07 (0.36–3.15)	Critically Low	Serious ROB, very serious inconsistency, serious imprecision	Low
He et al. 2023^15^	PGT‐A	No intervention	2	85/230	OR 1.51 (1.05–2.19)	Low	Serious ROB, serious inconsistency	Low

Abbreviations: CI, confidence interval; G‐CSF, granulocyte colony‐stimulating factor; GH, growth hormone; hCG, human chorionic gonadotropin; IVIG, intravenous immunoglobulins; Mixed, placebo and no intervention; NA, not applicable; NR, not reported; OR, odds ratio; PBMC, peripheral blood mononuclear cells; PGT‐A, preimplantation genetic testing for aneuploidy; PRP, platelet‐rich plasma; RCT, randomized clinical trial; ROB, risk of bias; RR, risk ratio.

^a^ Results from network meta‐analysis (NMA).

**TABLE 3:** Certainty of evidence for various interventions reported by meta‐analyses on the outcome: Live birth rate in the umbrella review of interventions for the treatment of recurrent embryo implantation failure.

Study ID	Intervention	Comparison	No. RCTs	No. of patients with LB/Total no.	Summary estimate (95% CI)	GRADE assessment	Reasons for downgrading	AMSTAR‐2 rating
Immunomodulatory interventions								
G‐CSF								
Hou et al. 2021^28^	G‐CSF	Placebo (2) or no intervention (1)	3	NR/372	RR, 1.43 (0.86–2.36)	Moderate	Serious inconsistency	Moderate
Wang et al. 2021^13^	G‐CSF	Placebo (2)	2	NR/320	OR, 1.36 (0.74–2.51)^a^	Low	Serious inconsistency, serious indirectness, serious imprecision	Critically low
Jin et al. 2022^11^	G‐CSF	Placebo (2)	2	NR/277	Mean 1.45 (0.7–2.97)^a^	Low	Very serious indirectness, serious inconsistency, serious imprecision	Critically low
Liu et al. 2022^12^	G‐CSF intrauterine	Placebo (2)	2	NR/411	OR, 3.81 (2.53–5.72)^a^	Moderate	NA	Critically Low
Kong et al. 2023^14^	G‐CSF	Placebo (3)	3	NR/413	OR, 0.75 (0.35–1.59)^a^	Low	Serious indirectness, serious inconsistency, serious imprecision	Low
Lu et al. 2023^26^	G‐CSF	Placebo (3)	3	87/320	RR, 1.51 (0.82–2.78)	Low	Serious ROB, serious inconsistency	High
He et al. 2023^15^	G‐CSF	Placebo (4)	4	NR/391	OR, 1.55 (0.78–3.07)^a^	Low	Serious ROB, serious inconsistency	Low
IVIG								
Achilli et al. 2018^46^	IVIG	Placebo (2)	2	14/90	OR, 2.1 (0.63–6.92)	Moderate	Serious imprecision	Critically low
GH								
Vera‐Montoya et al. 2023^27^	Growth hormone	No intervention (2)	2	34/112	OR, 5.13 (2.03–12.91)	High	NA	High
Intralipid								
Wang et al. 2021^13^	Intralipid	Placebo (1) or no intervention (1)	2	NR/244	OR, 2.04 (0.99–4.19)^a^	Low	Serious inconsistency, serious indirectness	Critically low
Zhou et al. 2020^43^	Intralipid	Placebo (2) or no intervention (2)	4	117/544	RR, 1.98 (1.39–2.8)	Moderate	Serious ROB	Low
Rimmer et al. 2021^45^	Intralipid	Placebo (3) or no intervention (2)	5	46/203	RR, 1.83 (1.42–2.35)	High	NA	Critically low
Kumar et al. 2021^53^	Intralipid	Placebo (2) or no intervention (3)	5	185/763	OR, 2.17 (1.54–3.05)	Low	Very serious ROB	Critically low
Han et al. 2021^32^	Intralipid	Placebo (3) or no intervention (2)	5	205/840	RR, 1.85 (1.44–2.38)	High	NA	Low
He et al. 2023^15^	Intralipid	No intervention (2)	2	NR/345	OR, 2.29 (0.92–5.54)^a^	Low	Serious ROB, serious inconsistency	Low
LMWH								
Liu et al. 2022^12^	LMWH	No intervention (2)	2	NR/357	OR, 3.75 (2.53–5.56)^a^	Moderate	Serious ROB	Critically Low
He et al. 2023^15^	LMWH	No intervention (4)	4	NR/538	OR, 1.31 (0.71–2.58)^a^	Low	Serious inconsistency, serious imprecision	Low
PBMC								
Wu et al. 2019^62^	PBMC	No intervention (2)	2	59/235	OR, 2.43 (1.32–4.49)	High	NA	Critically Low
Wang et al. 2021^13^	PBMC	Placebo (1) or no intervention (2)	3	NR/335	OR, 2.96 (1.67–5.27)^a^	Low	Serious inconsistency, serious indirectness	Critically Low
Jin et al. 2022^11^	PBMC	No intervention (2)	2	NR/312	OR, 2.55 (1.27–5.11)^a^	Moderate	Serious inconsistency	Critically Low
Liu et al. 2022^12^	PBMC	No intervention (2)	2	NR/567	OR, 8.07 (4.88–13.33)^a^	Low	Serious ROB, serious imprecision	Critically Low
Kong et al. 2023^14^	PBMC	No intervention (2)	2	NR/298	OR, 0.44 (0.14–1.35)^a^	Low	Serious ROB, serious inconsistency, serious indirectness, serious imprecision	Low
He et al. 2023^15^	PBMC	No intervention (4)	4	NR/389	OR, 3.29 (1.51–7.5)	Moderate	Serious inconsistency	Low
PRP								
Jin et al. 2022^11^	PRP	Placebo (2) or no intervention (1)	3	NR/658	OR, 5.96 (3.38–10.52)^a^	Moderate	Serious inconsistency	Critically Low
Liu et al. 2022^55^	PRP	Placebo (1) or no intervention (1)	2	93/473	OR, 11.02 (5.72–21.21)	Low	Serious ROB, serious imprecision	Critically Low
Hu et al. 2022^51^	PRP	Placebo (1) or no intervention (1)	2	91/433	RR, 7.03 (3.91–12.66)	Low	Very serious ROB, serious imprecision	Critically Low
Maged et al. 2023^35^	PRP	Placebo (1) or no intervention (1)	2	18/130	OR, 2.36 (0.15–36.35)	Low	Serious ROB, serious inconsistency, serious imprecision	Low
Anitua et al. 2023^25^	PRP	Placebo (2) or no intervention (1)	3	107/523	RR, 3.36 (0.84–13.45)	Low	Very serious inconsistency	High
Kong et al. 2023^14^	PRP	Placebo (1) or no intervention (1)	2	NR/593	OR, 0.27 (0.07–0.97)^a^	Low	Serious ROB, serious inconsistency, serious indirectness	Low
He et al. 2023^15^	PRP	Placebo (2) or no intervention (1)	3	NR/563	OR, 4.82 (2.13–9.95)^a^	Low	Serious ROB, serious inconsistency	Low
Uterine and endometrial interventions								
Hysteroscopy								
Mao et al. 2019^59^	Hysteroscopy	No intervention (2)	2	NR/1160	OR, 1.38 (0.8–2.38)	Moderate	Serious inconsistency	Critically low
He et al. 2023^15^	Hysteroscopy	No intervention (3)	3	NR/1480	OR, 1.58 (0.9–2.85)^a^	Low	Very serious Rob, serious inconsistency	Low
Intentional endometrial injury								
Potdar et al. 2012^36^	Intentional endometrial injury	No intervention (2)	2	131/525	RR, 2.63 (1.94–3.57)	High	NA	Low
Vitagliano et al. 2018^30^	Intentional endometrial injury	Placebo (1) or no intervention (6)	7	145/702	RR, 1.64 (1.21–2.21)	Moderate	Serious ROB	Moderate
Sar‐Shalom Nahshon et al. 2019^39^	Intentional endometrial injury	No intervention (3)	3	73/276	RR, 1.22 (0.52–2.82)	Low	Serious ROB, serious inconsistency, serious imprecision	Low
Busnelli et al. 2021^7^	Intentional endometrial injury	No intervention (3)	3	83/376	RR, 1.55 (0.81–2.94)	Moderate	Serious ROB	Low
Jin et al. 2022^11^	Intentional endometrial injury	No intervention (5)	5	NR/891	OR, 1.7 (1.07–2.69)^a^	Low	Serious inconsistency, very serious indirectness	Critically Low
Maged et al. 2023^44^	Intentional endometrial injury	No intervention (6)	6	189/707	OR, 0.95 (0.44–2.01)	Low	Very serious ROB, serious inconsistency, serious imprecision	Low
He et al. 2023^15^	Intentional endometrial injury	No intervention (3)	3	NR/396	OR, 1.55 (0.77–3.16)^a^	Low	Very serious ROB, serious inconsistency	Low
Laboratory interventions								
PGT‐A								
Busnelli et al. 2021^7^	PGT‐A	No intervention	2	75/230	RR 0.98 (0.32–2.94)	Critically low	Serious ROB, very serious inconsistency, serious imprecision	Low
He et al. 2023^15^	PGT‐A	No intervention	2	75/230	OR 1.01 (0.54–1.90)	Low	Serious ROB, serious inconsistency	Low

Abbreviations: CI, confidence interval; G‐CSF, granulocyte colony‐stimulating factor; GH, growth hormone; IVIG, intravenous immunoglobulins; LMWH, low molecular weight heparin; NA, not applicable; NR, not reported; OR, odds ratio; PBMC, peripheral blood mononuclear cells; PGT‐A, preimplantation genetic testing for aneuploidy; PRP, platelet‐rich plasma; RCTs, randomized clinical trials; RR, risk ratio; ROB, risk of bias.

^a^ Results from network meta‐analysis (NMA).

### 3.3 Effect of interventions

**FIGURE 3:**
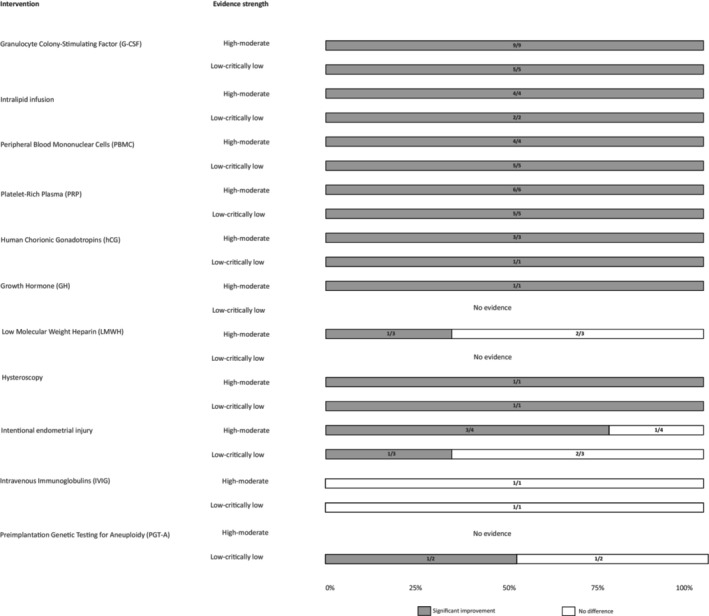
Change in clinical pregnancy rate (CPR) associated with different interventions for recurrent implantation failure grouped by evidence strength (GRADE assessment) of meta‐ analyses included in umbrella review. Data presented as 100% stacked bar chart with numbers inside bars indicating number of meta‐analyses with statistically significant improvements (color code: Gray) or no difference (color code: White) (see Table 2 for details of meta‐analysis).

**FIGURE 4:**
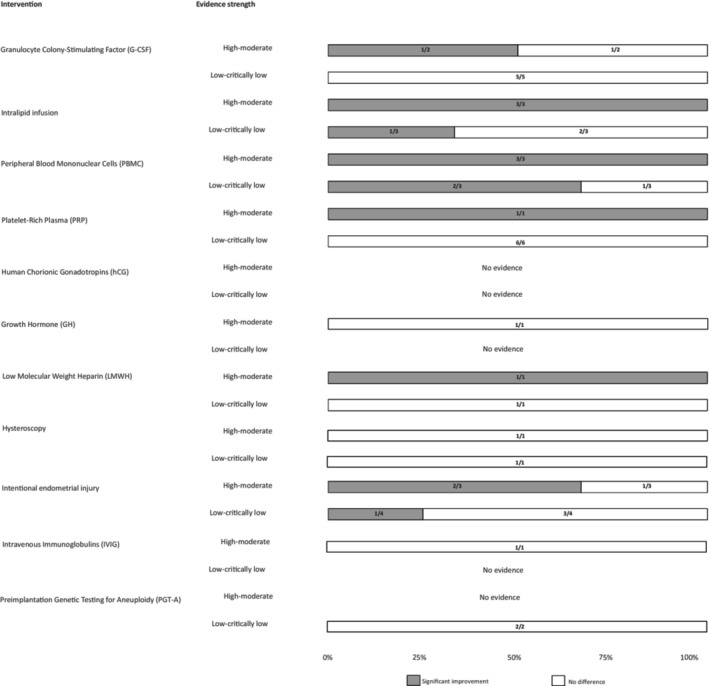
Change in live birth rate (LBR) associated with different interventions for recurrent implantation failure grouped by evidence strength (GRADE assessment) of meta‐analyses included in umbrella review. Data presented as 100% stacked bar chart with numbers inside bars indicating number of meta‐analyses with statistically significant improvements (color code: Gray) or no difference (color code: White) (see Table 3 for details of meta‐analysis).

The changes in CPR and LBR associated with different interventions for RIF grouped by evidence strength (GRADE assessment) are given in Figures 3 and 4. The evidence strength for CPR was high‐moderate in 36 MAs and low‐critically low in 23 MAs. The evidence strength for LBR was high or moderate in 17 MAs and low or critically low in 22 MAs. Granulocyte colony‐stimulating factor (G‐CSF) showed improvement in CPR in 13/14 (93%) MAs (9/13, 69.2% high‐moderate evidence strength) and in LBR in 1/7 (14%) MAs (moderate evidence strength). Intralipid infusion showed improvement in CPR in 6/6 (100%) MAs (4/6, 57.14% high‐moderate evidence strength) and in LBR in 4/6 (66.6%) MAs (3/4, 75% high‐moderate evidence strength). Peripheral blood mononuclear cells (PBMC) showed improvement in CPR in 8/9 (88%) MAs (4/8, 50% high‐moderate evidence strength) and in LBR in 5/6 (83.33%) MAs (3/5, 60% high‐moderate evidence strength). Platelet‐rich plasma (PRP) intervention showed improvement in CPR in 10/11 (91%) MAs (6/10, 60% high‐moderate evidence strength) and in LBR in 5/7 (71.42%) MAs (1/5, 20% moderate evidence strength). Human chorionic gonadotropins (hCG) showed improvement in CPR in 3/4 (75%) MAs (3/3 (100%) high‐moderate evidence strength). Growth hormone (GH) showed improvement in CPR in 1/1 (100%) MA (1/1, 100% high evidence strength) and in LBR in 1/1 (100%) MA (1/1, 100% high evidence strength). Low molecular weight heparin (LMWH) showed improvement in CPR in 1/3 (33.33%) MAs (1/1, 100% moderate evidence strength) and in LBR in 1/2 (50%) MAs (1/1, 100% moderate evidence strength). For uterine and endometrial interventions, hysteroscopy showed improvement in CPR in 2/2 (100%) MAs (1/2, 50% moderate evidence strength). Intentional endometrial injury showed improvement in CPR in 4/7 (57.14%) MAs (3/4, 75% high‐moderate evidence strength) and in LBR in 3/7 (42.86%) MAs (2/3, 66.66% high‐moderate evidence strength). For laboratory interventions, preimplantation genetic testing for aneuploidy (PGT‐A) showed improvement in CPR in 1/2 (50%) MAs (1/1, 100% low evidence strength), but not LBR.

## 4 DISCUSSION

### 4.1 Principal findings

This overview assessed 47 SRs, encompassing 375 RCTs, on the effectiveness of various interventions for RIF in terms of CPR and LBR. According to our search, this is the only comprehensive overview with methodological quality and evidence strength assessment focusing on interventions for RIF. Overall, among the 10 different interventions reviewed, we found that nine demonstrated statistically significant support for improving CPR, and seven showed similar results for LBR. Most of the significant meta‐analytical associations (32 out of 52 for CPR and 12 out of 16 for LBR) were of high or moderate evidence strength, as assessed by GRADE. Our review indicated that the majority of interventions for RIF involved immunomodulatory treatments, particularly G‐CSF, PRP, PBMC, and intralipid infusion. Eight interventions (G‐CSF, PBMC, PRP, GH, hCG, intralipid infusion, LMWH, and intentional endometrial injury) statistically demonstrated superiority over placebo or no intervention in enhancing clinical outcomes for patients with RIF.

For instance, while historical evidence suggested a consistent trend with considerable certainty regarding the benefits of G‐CSF and PRP in improving CPR, recent findings indicate no significant improvement. Similarly, although earlier studies showed a consistent trend with considerable certainty for Intralipid infusion in enhancing CPR and LBR, recent evidence offers only limited support for CPR improvement. For PBMC, evidence shows a converging and convincing trend supporting improvements in clinical outcomes. In contrast, there is limited evidence to support the use of GH, hCG, hysteroscopy, and LMWH for improving clinical outcomes. Although intentional endometrial injury was previously associated with a consistent trend and considerable certainty for improving clinical outcomes, recent findings indicate no significant improvement. As for PGT‐A, there is limited evidence showing no improvement in CPR and LBR; however, in clinical practice, it may be proposed as a pragmatic approach to address embryo aneuploidy in patients with advanced maternal age and RIF, in alignment with ESHRE recommendations.^9^ It is essential to consider adverse events associated with pharmacological administration when implementing or recommending treatments in clinical practice, to carefully balance the potential benefits and risks.

### 4.2 Strength and limitations

The review was reported according to established guidelines. We conducted a comprehensive search, updated during the course of our work, to capture the most current literature. We opted for an overview of reviews to help explore the heterogeneity in the populations, interventions, and outcomes studied. Our overview indicated the tendency or direction of the effect of interventions in RIF patients. We systematically appraised the evidence presented in the reviews. This work incorporated evidence from 98 MAs. To enhance transparency, we assessed the evidence of RCTs using the GRADE framework.

Our analysis primarily focused on summarizing the synthesized effects. In our appraisal of methodological quality, we did not re‐evaluate the original statistics reported, such as the *I* ^2^ metric for between‐study heterogeneity, nor did we reassess evidence for small‐study effects. This approach may limit our understanding of study variability and potential biases within the reviewed literature. When evaluating the results of the meta‐analyses in our umbrella review, it is crucial to note that the individual studies included were extensively scrutinized in the original reviews. Thus, our conclusions are based on the interpretations and summary estimates of pooled effects presented by the authors of these meta‐analyses. There was a notable degree of overlap among the RCTs included in the systematic reviews and meta‐analyses, indicating that numerous studies were repeatedly included across some meta‐analyses but not others. This repetition may inadvertently amplify the influence of certain studies in the umbrella review, a phenomenon attributable to the timeframe of the searches or the year of publication. It is anticipated that more recent meta‐analyses would incorporate newer studies while also encompassing earlier ones. Additionally, the variability in population characteristics, comparators, and outcome measures restricted the synthesis of results. Despite these limitations, this umbrella review stands as the first to systematically summarize current evidence regarding the effectiveness of interventions for RIF treatment in an umbrella review.

### 4.3 Comparison with current overviews and future recommendations

To our knowledge, this is the first umbrella review of its kind. This review emphasizes the challenges in evaluating evidence syntheses due to overlap and generally variable methodological quality, underscoring the need for higher‐quality intervention studies rather than the publication of additional meta‐analyses. Existing overviews, in the form of network meta‐analyses, present heterogeneous conclusions, ranging from recommendations for new RCTs to advocating for specific interventions.^11^, ^12^, ^13^, ^14^, ^15^ Such reviews tend to mix up placebo and no treatment when constructing the networks. Authors and readers are advised to consult the AMSTAR‐2 guidelines prior to conducting and using meta‐analyses for RIF interventions, as inferences depend heavily on the methodological quality of evidence syntheses. The existing network meta‐analyses often compare interventions with controls that mix placebos and no interventions together. Future network meta‐analyses should evaluate whether there is a placebo effect. If a placebo effect is present, future trials should ethically be required to consider using placebo instead of no treatment as a comparator. Overall, more rigorously designed primary studies are necessary to clearly demonstrate the effects of interventions on clinical outcomes in RIF patients and to further explore the distinct components that make interventions effective.

## 5 CONCLUSIONS

Immunomodulatory interventions such as G‐CSF, PRP, PBMC, intralipid infusion, and intentional endometrial injury could potentially enhance the management of RIF patients based on evidence of high to moderate strength. However, the heterogeneity due to variations in the definitions of RIF places provisos on the interpretation of these findings.

## AUTHOR CONTRIBUTIONS

*Conceptualization*: Abdulla Almohammadi, Fadi Choucair. *Data curation*: Abdulla Almohammadi, Fadi Choucair. *Formal analysis*: Abdulla Almohammadi, Fadi Choucair, Naomi Cano‐Ibáñez. *Supervision*: Aurora Bueno‐Cavanillas, Khalid S Khan. *Visualization*: Abdulla Almohammadi, Fadi Choucair, Aurora Bueno‐Cavanillas, Khalid S Khan. *Writing* – *original draft*: Abdulla Almohammadi, Fadi Choucair. *Review and editing*: Abdulla Almohammadi, Fadi Choucair, Aurora Bueno‐Cavanillas, Khalid S Khan, Naomi Cano‐Ibáñez.

## FUNDING INFORMATION

No funding was required or obtained for this research.

## CONFLICT OF INTEREST STATEMENT

The authors declare no conflicts of interest.

## Supporting information


**Data S1.** Supporting Information.

## Data Availability

Data sharing is not applicable to this article as no new data were created or analyzed in this study.
