# Supplementary material for: Interventions for recurrent embryo implantation failure: An umbrella review
Source: Int J Gynaecol Obstet. 2024 Dec 5;169(2):539–56. doi: 10.1002/ijgo.16066 (PMC12011071; doi:10.1002/ijgo.16066)
Supplement: Supplementary file 1 — Data S1. Supporting Information. [file IJGO-169-539-s001.docx]

**Appendix**

**Table S1. The 2020 PRISMA checklist for reporting a systematic review**

| **Section and Topic** | **Item #** | **Checklist item** | **Location where item**  **is reported** |
| --- | --- | --- | --- |
| **TITLE** | | |  |
| Title | 1 | Identify the report as a systematic review. | 1 |
| **ABSTRACT** | | |  |
| Abstract | 2 | See the PRISMA 2020 for Abstracts checklist. | 1 |
| **INTRODUCTION** | | |  |
| Rationale | 3 | Describe the rationale for the review in the context of existing knowledge. | 2 |
| Objectives | 4 | Provide an explicit statement of the objective(s) or question(s) the review addresses. | 3 |
| **METHODS** | | |  |
| Eligibility criteria | 5 | Specify the inclusion and exclusion criteria for the review and how studies were grouped for the syntheses. | 3 |
| Information sources | 6 | Specify all databases, registers, websites, organisations, reference lists and other sources searched or consulted to identify studies. Specify the date when each source was last searched or consulted. | 3 |
| Search strategy | 7 | Present the full search strategies for all databases, registers and websites, including any filters and limits used. | 3 |
| Selection process | 8 | Specify the methods used to decide whether a study met the inclusion criteria of the review, including how many reviewers screened each record and each report retrieved, whether they worked independently, and if applicable, details of automation tools used in the process. | 3 |
| Data collection process | 9 | Specify the methods used to collect data from reports, including how many reviewers collected data from each report, whether they worked independently, any processes for obtaining or confirming data from study investigators, and if applicable, details of automation tools used in the process. | 4 |
| Data items | 10a | List and define all outcomes for which data were sought. Specify whether all results that were compatible with each outcome domain in each study were sought (e.g. for all measures, time points, analyses), and if not, the methods used to decide which results to collect. | 4 |
|  | 10b | List and define all other variables for which data were sought (e.g. participant and intervention characteristics, funding sources). Describe any assumptions made about any missing or unclear information. | 4 |
| Study risk of bias assessment | 11 | Specify the methods used to assess risk of bias in the included studies, including details of the tool(s) used, how many reviewers assessed each study and whether they worked independently, and if applicable, details of automation tools used in the process. | 4 |
| Effect measures | 12 | Specify for each outcome the effect measure(s) (e.g. risk ratio, mean difference) used in the synthesis or presentation of results. | 4 |
| Synthesis methods | 13a | Describe the processes used to decide which studies were eligible for each synthesis (e.g. tabulating the study intervention characteristics and comparing against the planned groups for each synthesis (item #5)). | 4 |
|  | 13b | Describe any methods required to prepare the data for presentation or synthesis, such as handling of missing summary statistics, or data conversions. | NA |
|  | 13c | Describe any methods used to tabulate or visually display results of individual studies and syntheses. | NA |
|  | 13d | Describe any methods used to synthesize results and provide a rationale for the choice(s). If meta-analysis was performed, describe the model(s), method(s) to identify the presence and extent of statistical heterogeneity, and software package(s) used. | NA |
|  | 13e | Describe any methods used to explore possible causes of heterogeneity among study results (e.g. subgroup analysis, meta-regression). | NA |
|  | 13f | Describe any sensitivity analyses conducted to assess robustness of the synthesized results. | NA |
| Reporting bias assessment | 14 | Describe any methods used to assess risk of bias due to missing results in a synthesis (arising from reporting biases). | NA |
| Certainty assessment | 15 | Describe any methods used to assess certainty (or confidence) in the body of evidence for an outcome. | 4 |

| **Section and Topic** | **Item #** | **Checklist item** | **Location where item**  **is reported** |
| --- | --- | --- | --- |
| **RESULTS** | | |  |
| Study selection | 16a | Describe the results of the search and selection process, from the number of records identified in the search to the number of studies included in the review, ideally using a flow diagram. | 4 |
|  | 16b | Cite studies that might appear to meet the inclusion criteria, but which were excluded, and explain why they were excluded. | 4 |
| Study characteristics | 17 | Cite each included study and present its characteristics. | 5 |
| Risk of bias in studies | 18 | Present assessments of risk of bias for each included study. | 5-6 |
| Results of individual studies | 19 | For all outcomes, present, for each study: (a) summary statistics for each group (where appropriate) and (b) an effect estimate and its precision (e.g. confidence/credible interval), ideally using structured tables or plots. | 7-10 |
| Results of syntheses | 20a | For each synthesis, briefly summarise the characteristics and risk of bias among contributing studies. | 7-10 |
|  | 20b | Present results of all statistical syntheses conducted. If meta-analysis was done, present for each the summary estimate and its precision (e.g. confidence/credible interval) and measures of statistical heterogeneity. If comparing groups, describe the direction of the effect. | 7-10 |
|  | 20c | Present results of all investigations of possible causes of heterogeneity among study results. | NA |
|  | 20d | Present results of all sensitivity analyses conducted to assess the robustness of the synthesized results. | NA |
| Reporting biases | 21 | Present assessments of risk of bias due to missing results (arising from reporting biases) for each synthesis assessed. | NA |
| Certainty of evidence | 22 | Present assessments of certainty (or confidence) in the body of evidence for each outcome assessed. | 7-10 |
| **DISCUSSION** | | |  |
| Discussion | 23a | Provide a general interpretation of the results in the context of other evidence. | 11 |
|  | 23b | Discuss any limitations of the evidence included in the review. | 13 |
|  | 23c | Discuss any limitations of the review processes used. | NA |
|  | 23d | Discuss implications of the results for practice, policy, and future research. | 13 |
| **OTHER INFORMATION** | | |  |
| Registration and protocol | 24a | Provide registration information for the review, including register name and registration number, or state that the review was not registered. | 2-3 |
|  | 24b | Indicate where the review protocol can be accessed, or state that a protocol was not prepared. | NA |
|  | 24c | Describe and explain any amendments to information provided at registration or in the protocol. | NA |
| Support | 25 | Describe sources of financial or non-financial support for the review, and the role of the funders or sponsors in the review. |  |
| Competing interests | 26 | Declare any competing interests of review authors. |  |
| Availability of data, code and other materials | 27 | Report which of the following are publicly available and where they can be found: template data collection forms; data extracted from included studies; data used for all analyses; analytic code; any other materials used in the review. |  |

*Source:* Page MJ, McKenzie JE, Bossuyt PM, Boutron I, Hoffmann TC, Mulrow CD, et al. The PRISMA 2020 statement: an updated guideline for reporting meta-analyses. BMJ 2021;372:n71. doi: 10.1136/bmj.n71 (65)

**Table S2. List of excluded studies**

| **No.** | **Title** | **DOI** | **Reason for rejection** |  |
| --- | --- | --- | --- | --- |
| 1 | Current approaches to diagnosing and treating endometrial receptor apparatus disorders in patients with recurrent implantation failures | 10.18565/aig.2021.3.26-35 | Irrelevant article |  |
| 2 | Antikoagulation und assistierte Reproduktion | 10.1007/s10304-008-0294-1 | Irrelevant article |  |
| 3 | Intrauterine administration of human chorionic gonadotropin (hCG) for subfertile women undergoing assisted reproduction. | 10.1002/14651858.CD011537.pub2 | Subfertile women only |  |
| 4 | Endometrial injection of embryo culture supernatant for subfertile women in assisted reproduction. | 10.1002/14651858.CD013063.pub2 | Subfertile women undergoing ART |  |
| 5 | Efficacy of intrauterine perfusion of peripheral blood mononuclear cells (PBMC) for infertile women before embryo transfer: meta-analysis. | 10.1080/01443615.2019.1673711 | Infertile women only |  |
| 6 | Antiphospholipid antibodies in women with recurrent embryo implantation failure: A systematic review and meta-analysis. | 10.1016/j.autrev.2022.103101 | No RCT in the review |  |
| 7 | Does Intrauterine Injection of hCG Improve IVF Outcome? A Systematic Review and a Meta-Analysis. | 10.3390/ijms232012193 | Target population was strictly not having RIF |  |
| 8 | Effect of sildenafil citrate on treatment of infertility in women with a thin endometrium: a systematic review and meta-analysis | 10.1177/0300060520969584 | Target population was strictly not having RIF |  |
| 9 | The effect of peri-implantation administration of uterine relaxing agents in assisted reproduction treatment cycles: a systematic review and meta-analysis. | 10.1016/j.rbmo.2016.01.004 | Target population was strictly not having RIF |  |
| 10 | The efficacy of intrauterine injection of human chorionic gonadotropin before embryo transfer in assisted reproductive cycles: Meta-analysis. | 10.1177/0300060515592903 | Target population was strictly not having RIF |  |
| 11 | Effect of assisted hatching on pregnancy outcomes: a systematic review and meta-analysis of randomized controlled trials | 10.1038/MAep31228 | Target population was strictly not having RIF |  |
| 12 | Impact of endometrial scratching on reproductive outcome in patients: A systematic review and meta-analysis. | 10.1097/MD.0000000000030150 | Target population was strictly not having RIF |  |
| 13 | Aspirin for Endometrial Preparation in Patients Undergoing IVF: A Systematic Review and Meta-analysis. | 10.1016/j.jogc.2021.03.018 | Target population was strictly not having RIF |  |
| 14 | Whether G-CSF administration has beneficial effect on the outcome after assisted reproductive technology? A systematic review and meta-analysis. | 10.1186/s12958-016-0197-2 | Target population was strictly not having RIF |  |
| 15 | Effectiveness of the use of Low Molecular Heparin in patients with repetition abortion history: Systematic review and meta-analysis | 10.5935/1518-0557.20200042 | Patients with repeated abortions (not implantation failure) |  |
| 16 | Intravenous immunoglobulin treatment for repeated IVF/ICSI failure and unexplained infertility: a systematic review and a meta-analysis. | 10.1111/aji.12170 | The review included only case-control studies |  |
| 17 | Evidence for the effectiveness of immunologic therapies in women with subfertility and/or undergoing assisted reproduction. | 10.1016/j.fertnstert.2022.04.015 | Target population was not strictly women with RIF |  |
| 18 | A meta-analysis of atosiban supplementation among patients undergoing assisted reproduction. | 10.1007/s00404-017-4455-0 | Target population was not strictly women with RIF |  |
| 19 | Unexplained Recurrent Miscarriage and Recurrent Implantation Failure: Is There a Place for Immunomodulation? | 10.1111/aji.12493 | Target population was not strictly women with RIF |  |
| 20 | Use of zona pellucida-bound spermatozoa as a natural selection in improvement of ICSI outcomes: A systematic review and meta-analysis | 10.1111/and.14022 | Participants? Not having RIF. Studies RCTs or not? |  |
| 21 | Proportion of Cytotoxic Peripheral Blood Natural Killer Cells and T-Cell Large Granular Lymphocytes in Recurrent Miscarriage and Repeated Implantation Failure: Case-Control Study and Meta-analysis | 10.1007/s00005-019-00546-5 | Case-control study and metanalysis, no treatment given |  |
| 22 | Lymphocyte immunotherapy in recurrent miscarriage and recurrent implantation failure. | 10.1111/aji.13408 | Review article only. No methods section |  |
| 23 | Antimüllerian hormone as predictor of implantation and clinical pregnancy after assisted conception: a systematic review and meta-analysis. | 10.1016/j.fertnstert.2014.09.041 | Review included observational studies, no outcome recorded |  |
| 24 | Correction to: Evaluating interventions and adjuncts to optimize pregnancy outcomes in subfertile women: an overview review. | 10.1093/humupd/dmac020 | Correction article of a review article |  |
| 25 | The effects of hydroxychloroquine on pregnancy outcomes in infertile women: a systematic review and meta-analysis | 10.25122/jml-2022-0095 | The review did not include any RCT |  |
| 26 | Progesterone supplementation to prevent recurrent miscarriage and to reduce implantation failure in assisted reproduction cycles | 10.1016/s1472-6483(10)62015-9 | No RCT included |  |
| 27 | Is there evidence to support serum antinuclear antibodies testing in women with recurrent implantation failure undergoing in vitro fertilization? | 10.1080/14647273.2017.1306657 | Commentary |  |
| 28 | Presence of antiphospholipid antibodies is associated with increased implantation failure following in vitro fertilization technique and embryo transfer: A systematic review and meta-analysis. | 10.1371/journal.pone.0260759 | Irrelevant review, no RCT, no outcome recorded |  |
| 29 | PAI-1 4G/4G Genotype Is Associated with Recurrent Implantation Failure: a Systematic Review and Meta-analysis. | 10.1007/s43032-021-00623-1 | No treatment given to patients |  |
| 30 | The Impact of New Immunological Therapeutic Strategies on Recurrent Miscarriage and Recurrent Implantation Failure | 10.1016/j.imlet.2021.05.008 | Review article, not a systematic review |  |
| 31 | Association between MTHFR polymorphisms (MTHFR C677T, MTHFR A1298C) and recurrent implantation failure: a systematic review and meta-analysis. | 10.1007/s00404-020-05851-5 | No treatment given to patients |  |
| 32 | Effects of chronic endometritis therapy on in vitro fertilization outcome in women with repeated implantation failure: a systematic review and meta-analysis | 10.1016/j.fertnstert.2018.03.017 | All studies were observational (three prospective and two retrospective |  |
| 33 | Polymorphisms of vascular endothelial growth factor and recurrent implantation failure: a systematic review and meta-analysis. | 10.1007/s00404-021-06072-0 | no treatment given. Showed association between polymorphisms of vascular endothelial growth factor and recurrent implantation failure: |  |
| 34 | Interventions for improving reproductive outcomes in women with recurrent implantation failure undergoing assisted reproductive techniques | 10.1002/14651858.CD010795 | Protocol article |  |
| 35 | Number and function of uterine natural killer cells in recurrent miscarriage and implantation failure: a systematic review and meta-analysis. | 10.1093/humupd/dmac006 | No treatment given to patients |  |
| 36 | Embryo transfer strategy and therapeutic options in infertile patients with thin endometrium: a systematic review. | 10.1007/s10815-019-01576-w | Target population was not strictly women with RIF but those with thin endometrium |  |
| 37 | The effectiveness of IVIG therapy in pregnancy and live birth rate of women with recurrent implantation failure (RIF): A systematic review and meta-analysis. | 10.1016/j.jri.2019.07.006 | This review did not include any RCT |  |
| 38 | Efficacy of intrauterine perfusion of granulocyte colony-stimulating factor (G-CSF) for Infertile women with thin endometrium: A systematic review and meta-analysis. | 10.1111/aji.12701 | Target population was not strictly women with RIF but those with thin endometrium |  |
| 39 | Remedies for recurrent implantation failure. | 10.1055/s-0034-1375182 | Review article, not a systematic review |  |
| 40 | Natural killer cells in female infertility and recurrent miscarriage: a systematic review and meta-analysis | 10.1093/humupd/dmt056 | Irrelevant review. No treatment given to patients |  |
| 41 | Comparison of pregnancy outcomes after vitrification at the cleavage and blastocyst stage: a meta-analysis | 10.1007/s10815-017-1040-1 | This review did not include any RCT |  |
| 42 | Metabolomic markers of biological fluid in women with reproductive failure: a systematic review of current literatures | 10.1093/biolre/ioac038 | Irrelevant review. No treatment given to patients |  |
| 43 | Impact of antibiotic treatment for chronic endometritis on pregnancy outcomes in women with reproductive failures (RIF and RPL): A systematic review and meta-analysis. | 10.3389/fmed.2022.980511 | The review did not include any RCT |  |
| 44 | Endometrial receptivity array before frozen embryo transfer cycles: a systematic review and meta-analysis. | 10.1016/j.fertnstert.2022.11.012 | Included patients in review were not those suffering from RIF |  |
| 45 | Opening the black box: why do euploid blastocysts fail to implant? A systematic review and meta-analysis. | 10.1093/humupd/dmad010 | Irrelevant review |  |
| 46 | Personalized embryo transfer guided by endometrial receptivity analysis: a systematic review with meta-analysis. | 10.1093/humrep/dead098 | Irrelevant review |  |
| 47 | The Effects of Acupuncture on Pregnancy Outcomes of Recurrent Implantation Failure: A Systematic Review and Meta-Analysis | 10.1155/2021/6661235 | Accupuncture is not a medical intervention |  |
| 48 | Immunotherapy to improve pregnancy outcome in women with abnormal natural killer cell levels/activity and recurrent miscarriage or implantation failure: A systematic review and meta-analysis | 10.1016/j.jri.2020.103189 | No RCTs reported data on RIF | |

**Table S3 Characteristics of the included meta-analyses**

| **Author, Year** | **Last year of search** | **Participants’ definition** | **Intervention** | **Comparators** | **Reported outcomes** | **No. of RCTs** |
| --- | --- | --- | --- | --- | --- | --- |
| *Immunomodulatory interventions* | | | | | | |
| Mohan S. Kamath, 2020 (24) | Till February 2019 | Women with RIF undergoing ART | G-CSF | Placebo | Clinical pregnancy rate | 7 |
| Ling Zhang, 2018 (43) | Till July 2017 | Infertile women undergoing ART | G-CSF | Placebo or no intervention | Clinical pregnancy rate | 10 |
| Ying Jiang, 2020 (53) | Till November 2019 | Women with unexplained RIF, at least 3 times | G-CSF | placebo or no intervention | Clinical pregnancy rate | 8 |
| Caiyun Wang, 2021 (13) | January, 2021 | RIF patients with at least one or more in vitro fertilization/embryo transfer (IVF/ ET) failure history | Immunotherapy (PBMC, G-CSF, PRP, sirolimus, recombinant Human leukemia inhibitory factor) | placebo or no intervention | Clinical pregnancy rate, Live birth rate | 2 |
| Zhijin Hou, 2021 (28) | Till October 2020 | Women under the age of 40 years with at least four implantation failures | G-CSF | placebo or no intervention | Clinical pregnancy rate, Live birth rate, | 12 |
| Mengqi Liu, 2022 (12) | Till August 2021 | Women with two or more episodes of implantation failure | immunomodulatory therapy | placebo or no intervention or standard care | Clinical pregnancy rate, Live birth rate, | 3 |
| Fu Lu, 2023 (26) | Till December 2021 | Women undergoing IVF with repeated implantation failure who were given G-CSF | G-CSF | placebo or no intervention | Clinical pregnancy rate, Live birth rate, | 5 |
| Vera-Montoya, 2023 (27) | Till August 2021 | Women with RIF undergoing ART | Growth hormone intervention | placebo or no intervention | Clinical pregnancy rate, Live birth rate, | 7 |
| Hebin Xie, 2019 (64) | January, 2019 | Women with two or more implantation failures | Human chorionic gonadotropin (hCG) | No intervention | Clinical pregnancy rate, Live birth rate, | 8 |
| Ping Zhou, 2020 (44) | Till 5 August 2019 | Women with at least one implantation failure undergoing IVF/ICSI cycles | Intralipid infusion | placebo or no intervention | Clinical pregnancy rate, Live birth rate, | 4 |
| E Jung Han, 2021 (32) | March, 2020 | Women with repeated implantation failure | Intralipid infusion | Placebo or no intervention | Clinical pregnancy rate, Live birth rate, | 3 |
| Michael P. Rimmer, 2021 (46) | Till September 2020 | Women with history of RIF | Intralipid infusion | placebo or no intervention | Clinical pregnancy rate, Live birth rate, | 6 |
| Parijot Kumar, 2021 (54) | 2020 | Women with RIF ± IVF/ICSI treatment | Intralipid infusion | placebo or no intervention | Clinical pregnancy rate, Live birth rate, | 6 |
| Yang Wu, 2019 (63) | April, 2018 | Women with RIF undergoing ART (fresh or frozen cycles) | PBMC | placebo or no intervention | Clinical pregnancy rate, Live birth rate, | 2 |
| Arezoo Maleki-Hajiagha, 2020 (59) | May, 2019 | Women with RIF | PRP | placebo or no intervention | Clinical pregnancy rate, Live birth rate, | 21 |
| Zahra Pourmoghadam, 2020 (29) | July, 2018 | Women between 30–45 years old who experienced at least three implantation failures | PBMC | No intervention | Clinical pregnancy rate, Live birth rate, | 2 |
| Kepeng Liu, 2022 (56) | 2022 | Infertile women who experienced one or more implantation failures | PRP | placebo or no intervention | Clinical pregnancy rate, Live birth rate, | 5 |
| Muzi Li, 2022 (35) | NR | Women with unexplained RIF | PRP | No intervention or other intervention | Clinical pregnancy rate, Live birth rate, | 1 |
| Shifu Hu, 2023 (52) | June, 2022 | Women with implantation failure undergoing treatment with assisted reproductive technology (ART) | PRP | placebo or no intervention | Clinical pregnancy rate, Live birth rate, | 3 |
| Ahmed M. Maged, 2023 (36) | August, 2022 | Women with RIF | PRP | placebo or no intervention or granulocyte colony-stimulating factor (G-CSF) | Clinical pregnancy rate, Live birth rate, | 1 |
| Eduardo Anitua, 2023 (25) | August ,2022 | Women undergoing assisted reproduction with a history of embryo transfer failure | PRP | placebo or no intervention | Clinical pregnancy rate, Live birth rate, | 2 |
| Arezoo Maleki-Hajiagha, 2019 (58) | April, 2018 | Women undergoing ART with three or more previous implantation failures | PBMC | No intervention | Clinical pregnancy rate, Live birth rate, | 22 |
| Chiara Achilli, 2018 (47) | NR | Women with RIF | Immunotherapy | Placebo or no intervention | Live birth rate | 6 |
| Haiyu Deng, 2022 (31) | August, 2022 | Infertile women with RIF who had experienced at least three implantation failures | PRP | Standard care or HRT | Clinical pregnancy rate, Live birth rate, | 2 |
| Kayhan Yakin, 2019 (42) | April, 2017 | Women with RIF undergoing ART | PBMC | No intervention | Clinical pregnancy rate, Live birth rate, | 46 |
| Marcelo Borges Cavalcante , 2020 (49) | NR | Women with RIF undergoing ART | PRP, PBMC, G-CSF, HCG | Mixed comparators | Clinical pregnancy rate | - |
| Mohan S. Kamath, 2017 (33) | January, 2016 | subfertile women undergoing ART with RIF | G-CSF | placebo or no intervention | Clinical pregnancy rate, Live birth rate, | 2 |
| Mylena Naves de Castro Rocha, 2020 (51) | March, 2018 | Women with repeated implantation failure associated with thin endometrium | G-CSF | NR | Clinical pregnancy rate | 21 |
| Neelam Potdar, 2013 (38) | 2012 | Women with RIF undergoing IVF/ICSI | LMWH | placebo or no intervention | Clinical pregnancy rate, Live birth rate, | 2 |
| Qi Qin, 2021 (39) | October, 2020 | Women undergoing assisted reproductive technology (ART) who had experienced RIF | PBMC | placebo or no intervention | Clinical pregnancy rate, Live birth rate, | 13 |
| Wael Saab, 2021 (62) | NR | Women with RIF | immunoglobulin G treatment (intravenous) | NR | Clinical pregnancy rate, Live birth rate, | 3 |
| Xinliang Kong, 2023 (34) | Till 16 April 2021 | women who experienced at least two implantation failures | hCG, G-CSF, PBMCs and PRP | No intervention | Clinical pregnancy rate, Live birth rate, | 6 |
| *Uterine and endometrial interventions* | | | | | | |
| Xiaoyan Mao, 2019 (60) | May, 2017 | women with at least 2 failed IVF–embryo transfer attempts | Hysteroscopy | No intervention | Clinical pregnancy rate, Live birth rate, | 6 |
| Neelam Potdar, 2012 (37) | 2012 | Women with RIF undergoing/ICSI treatment | Endometrial injury (endometrial biopsy/scratch and/or hysteroscopy) | No intervention | Clinical pregnancy rate, Live birth rate, | 9 |
| Amerigo Vitagliano, 2018 (30) | NR | Infertile women undergoing IVF after one or more failed embryo transfers (ET) | Endometrial scratch injury | placebo or no intervention | Clinical pregnancy rate, Live birth rate, | 4 |
| Chen Sar-Shalom Nahshon, 2019 (40) | NR | Women with at least one previous failed IVF cycle | Endometrial injury | no intervention | Clinical pregnancy rate, Live birth rate | 5 |
| Hanyu Cao, 2018 (48) | 2016 | Women with at least 2 implantation failures | Hysteroscopy | NR | Clinical pregnancy rate, Live birth rate | 5 |
| Nikoletta Panagiotopoulou, 2015 (61) | Till 30 May, 2015 | Women undergoing ART with 2 or more previous implantation failures | Endometrial injury | placebo or no intervention | Clinical pregnancy rate, Live birth rate | 21 |
| Qian-YiHuang, 2017 (66) | January, 2017 | Women undergoing IVF | Atosiban | Placebo | Clinical pregnancy rate | 1 |
| Ruxin Wang, 2023 (41) | December, 2022 | Women undergoing IVF-ET who had experienced RIF | Atosiban | placebo or no intervention | Clinical pregnancy rate, Live birth rate | 2 |
| Xueying Li, 2023 (55) | January, 2022 | Women with RIF | Gonadotropin Release Hormone (GnRH) | placebo or no intervention | Clinical pregnancy rate | 1 |
| Ahmed M. Maged, 2023 (45) | April, 2023 | Women with two or more IVF failures | Intentional endometrial injury | Placebo or no intervention | Clinical pregnancy rate, Live birth rate | 9 |
| *Interventions based on diagnostic findings* |  |  |  |  |  |  |
| Xinghan Cheng, 2022 (50) | January, 2022 | RIF patients (defined as at least two previous failed IVF-ET attempts) with or without histologically confirmed CE | Antibiotic therapy | No intervention | Clinical pregnancy rate, Live birth rate | 1 |
| *Mixed interventions* |  |  |  |  |  |  |
| Antonis Makrigiannakis, 2021 (57) | NR | Women with RIF | Endometrial injury, HCG, PBMC, PRP, G-CSF, Growth Hormone, Atosiban, Antibiotics | No intervention | Clinical pregnancy rate | NR |
| Xin HangJin, 2022 (11) | Till 1 May, 2022 | Women with two or more implantation failures undergoing fresh or frozen embryo transfer (ET). | Endometrial scratching, G-CSF, HCG, PBMC, PRP | placebo or no intervention | Clinical pregnancy rate, Live birth rate, | 21 |
| Andrea Busnelli, 2021 (7) | May, 2020 | Women with RIF (at least three ET attempts) | Intentional Endometrial injury, Hysteroscopy, Atosiban, Sequential embryo transfers, preimplantation genetic testing for aneuploidy (PGT-A), Blastocyst-stage ET, assisted hatching, G-CSF, intravenous intralipid infusion, low molecular weight heparin (LMWH), peripheral blood mononuclear cells (PBMC), PRP, | placebo or no intervention | Clinical pregnancy rate, Live birth rate | 3 |
| Yunan He, 2023 (15) | August, 2022 | Women with two or more IVF failures | Hysteroscopy, HCG, PBMC, PRP, G-CSF, LMWH, intralipid, PGT-A | Placebo or no intervention | Clinical pregnancy rate, Live birth rate | 45 |

(ET=embryo transfer; G-CSF= Granulocyte Colony-Stimulating Factor; GnRH= gonadotropin releasing hormone; hCG=human chorionic gonadotropin, HRT=hormone replacement therapy, IVIG=intravenous immunoglobulins, LMWH=low molecular weight heparin; NR=not reported; PGT-A=preimplantation genetic testing for aneuploidy; PRP=Platelet-Rich Plasma; PBMC= Peripheral Blood Mononuclear Cells; RIF=recurrent implantation failure).

**Table S4. AMSTAR2 scores of included meta-analyses.**

|  | **Author, Year** | **1** | **2*** | **3** | **4*** | **5** | **6** | **7*** | **8** | **9*** | **10** | **11*** | **12** | **13*** | **14** | **15*** | **16** | **Yes** | **Partial Yes** | **No** | **Overall quality** |
| --- | --- | --- | --- | --- | --- | --- | --- | --- | --- | --- | --- | --- | --- | --- | --- | --- | --- | --- | --- | --- | --- |
| 1 | Mohan S Kamath, 2020 ^(24)^ | Y | N | Y | Y | Y | Y | Y | Y | Y | Y | Y | Y | Y | Y | Y | Y | 15 | - | 1 | Low |
| 2 | Ling Zhang, 2018 ^(43)^ | Y | Y | Y | PY | Y | Y | N | Y | Y | N | Y | Y | Y | Y | Y | Y | 13 | 1 | 2 | Low |
| 3 | Ying Jiang, 2020 ^(53)^ | Y | N | N | PY | Y | Y | N | PY | Y | Y | Y | Y | Y | Y | Y | Y | 11 | 2 | 3 | Critically Low |
| 4 | Caiyun Wang, 2021 ^(13)^ | Y | N | N | Y | Y | Y | N | PY | Y | Y | Y | Y | Y | Y | Y | Y | 12 | 1 | 3 | Critically Low |
| 5 | Zhijin Hou, 2021 ^(28)^ | Y | Y | N | PY | Y | Y | Y | Y | Y | N | Y | Y | Y | Y | N/A | Y | 12 | 1 | 3 | Moderate |
| 6 | Mengqi Liu, 2022 ^(12)^ | Y | N | N | PY | Y | Y | Y | PY | Y | N | Y | N | N | Y | Y | Y | 9 | 2 | 5 | Critically Low |
| 7 | Fu Lu, 2023^(26)^ | Y | Y | Y | PY | Y | Y | Y | Y | Y | N | Y | Y | Y | Y | Y | Y | 14 | 1 | 1 | High |
| 8 | Vera-Montoya, 2023 ^(27)^ | Y | Y | Y | PY | Y | Y | Y | PY | Y | N | Y | Y | Y | Y | N/A | Y | 12 | 2 | 2 | High |
| 9 | Hebin Xie, 2019 ^(64)^ | Y | N | N | PY | Y | Y | N | PY | Y | N | Y | Y | Y | Y | N/A | Y | 9 | 2 | 5 | Critically Low |
| 10 | Ping Zhou, 2020 ^(44)^ | Y | Y | N | Y | Y | Y | N | PY | Y | N | Y | Y | Y | Y | Y | Y | 12 | 1 | 3 | Low |
| 11 | E Jung Han, 2021 ^(32)^ | Y | Y | Y | PY | Y | Y | N | PY | Y | N | Y | Y | Y | Y | Y | Y | 12 | 2 | 2 | Low |
| 12 | Michael P. Rimmer,2021 ^(46)^ | Y | Y | N | Y | Y | Y | N | PY | Y | N | Y | Y | Y | Y | Y | Y | 12 | 1 | 3 | Low |
| 13 | Parijot Kumar, 2021 ^(54)^ | Y | N | N | Y | Y | Y | N | PY | N | N | Y | N | N | Y | N | Y | 7 | 1 | 8 | Critically Low |
| 14 | Yang Wu, 2019 ^(63)^ | Y | N | Y | PY | Y | Y | N | PY | Y | N | Y | Y | Y | Y | N/A | Y | 10 | 2 | 4 | Critically Low |
| 15 | Arezoo Maleki-Hajiagha, 2020 ^(59)^ | Y | N | Y | Y | Y | Y | Y | PY | Y | N | Y | Y | N | Y | Y | Y | 12 | 1 | 3 | Critically Low |
| 16 | Zahra Pourmoghadam, 2020 ^(29)^ | Y | Y | N | PY | Y | Y | Y | PY | Y | N | Y | Y | Y | Y | N/A | Y | 11 | 2 | 2 | Moderate |
| 17 | Kepeng Liu, 2022 ^(56)^ | Y | N | N | PY | Y | Y | N | PY | Y | N | Y | Y | Y | Y | N | Y | 9 | 2 | 4 | Critically Low |
| 18 | Muzi Li, 2022 ^(35)^ | Y | N | N | PY | Y | Y | Y | Y | Y | N | Y | Y | Y | Y | Y | Y | 12 | 1 | 3 | Low |
| 19 | Shifu Hu, 2023 ^(67)^ | Y | N | N | PY | Y | Y | N | Y | Y | N | Y | Y | Y | Y | Y | Y | 11 | 1 | 4 | Critically Low |
| 20 | Ahmed M. Maged, 2023 ^(36)^ | Y | Y | N | PY | Y | Y | N | PY | Y | N | Y | Y | Y | Y | Y | Y | 12 | 2 | 2 | Low |
| 21 | Eduardo Anitua, 2023 ^(25)^ | Y | Y | N | PY | Y | Y | Y | Y | Y | Y | Y | Y | Y | Y | N/A | Y | 13 | 1 | 2 | High |
| 22 | Arezoo Maleki-Hajiagha, 2019 ^(58)^ | Y | Y | N | Y | Y | Y | N | Y | Y | Y | Y | Y | Y | Y | N | Y | 13 | 0 | 3 | Critically Low |
| 23 | Chiara Achilli, 2018 ^(47)^ | Y | N | N | PY | Y | Y | Y | Y | Y | N | Y | N | N | Y | Y | Y | 9 | 2 | 5 | Critically Low |
| 24 | Haiyu Deng, 2022 ^(31)^ | Y | Y | N | PY | Y | Y | N | PY | Y | N | Y | Y | Y | Y | Y | Y | 11 | 2 | 3 | Low |
| 25 | Kayhan Yakin, 2019 ^(42)^ | Y | N | N | Y | Y | Y | Y | PY | Y | N | Y | Y | Y | Y | N/A | Y | 11 | 1 | 4 | Low |
| 26 | Marcelo Borges Cavalcante, 2020 ^(49)^ | N | N | N | N | Y | Y | N | PY | N | N | N/A | N/A | N/A | N | N/A | N | 2 | 1 | 13 | Critically Low |
| 27 | Mohan S Kamath, 2017 (33) | Y | Y | N | Y | Y | Y | N | PY | Y | N | Y | Y | Y | Y | N/A | Y | 11 | 1 | 4 | Low |
| 28 | Mylena Naves de Castro Rocha, 2020 ^(51)^ | N | N | N | PY | Y | Y | N | N | N | N | N/A | N/A | N/A | N | N/A | Y | 3 | 1 | 12 | Critically Low |
| 29 | Neelam Potdar, 2013 ^(38)^ | Y | N | Y | PY | Y | Y | Y | PY | Y | N | Y | Y | Y | Y | Y | Y | 12 | 2 | 2 | Low |
| 30 | Qi Qin, 2021 ^(39)^ | Y | Y | N | PY | N | Y | N | Y | Y | N | Y | Y | Y | Y | N/A | Y | 10 | 1 | 5 | Low |
| 31 | Wael Saab, 2021 ^(62)^ | Y | N | N | N | N | N | N | PY | N | N | N/A | N/A | N | N | N | N | 1 | 1 | 14 | Critically Low |
| 32 | Xinliang Kong, 2023 ^(34)^ | Y | Y | N | PY | Y | Y | N | PY | Y | N | Y | Y | Y | Y | Y | Y | 11 | 2 | 3 | Low |
| 33 | Xiaoyan Mao, 2019 ^(60)^ | Y | N | N | PY | Y | N | N | PY | Y | N | Y | Y | Y | Y | N/A | Y | 8 | 2 | 6 | Critically Low |
| 34 | Neelam Potdar, 2012 ^(37)^ | Y | N | Y | PY | Y | Y | Y | N | Y | N | Y | Y | Y | Y | Y | N | 11 | 1 | 4 | Low |
| 35 | Amerigo Vitagliano, 2018 ^(30)^ | Y | Y | N | PY | Y | Y | Y | Y | Y | N | Y | Y | Y | Y | Y | Y | 13 | 1 | 2 | Moderate |
| 36 | Chen Sar-Shalom Nahshon, 2019 ^(40)^ | Y | Y | N | PY | Y | Y | Y | Y | Y | N | Y | Y | Y | Y | N | Y | 13 | 1 | 3 | Low |
| 37 | Hanyu Cao, 2018 ^(48)^ | Y | N | N | PY | Y | Y | N | PY | Y | N | Y | Y | Y | Y | Y | Y | 10 | 2 | 4 | Critically Low |
| 38 | Nikoletta Panagiotopoulou, 2015 ^(61)^ | Y | N | N | Y | Y | Y | N | Y | Y | N | N/A | N/A | Y | Y | N/A | Y | 9 | 0 | 7 | Critically Low |
| 39 | Qian-Yi Huang, 2017 ^(66)^ | Y | N | N | PY | Y | Y | N | PY | Y | N | Y | Y | Y | N | N | Y | 8 | 2 | 6 | Critically Low |
| 40 | Ruxin Wang, 2023 ^(41)^ | Y | Y | Y | PY | Y | Y | N | PY | Y | N | Y | Y | Y | Y | Y | Y | 12 | 2 | 2 | Low |
| 41 | Xueying Li, 2023 ^(68)^ | Y | N | Y | PY | Y | Y | N | PY | Y | N | Y | N | N | N | N | Y | 7 | 2 | 7 | Critically Low |
| 42 | Xinghan Cheng, 2022 ^(50)^ | Y | N | N | N | Y | Y | N | PY | Y | N | Y | Y | Y | Y | N/A | Y | 9 | 1 | 6 | Critically Low |
| 43 | Antonis Makrigiannakis, 2021 ^(57)^ | N | N | N | N | N | N | N | N | N | N | N/A | N/A | N | Y | N | N | 1 | 0 | 15 | Critically Low |
| 44 | Xin HangJin, 2022 ^(11)^ | Y | N | N | PY | Y | Y | N | Y | Y | N | Y | Y | Y | Y | N | Y | 10 | 1 | 5 | Critically Low |
| 45 | Andrea Busnelli, 2021 ^(7)^ | Y | N | N | PY | Y | Y | Y | PY | Y | N | Y | Y | Y | Y | Y | Y | 11 | 2 | 3 | Low |
| 46 | Ahmed M. Maged, 2023 ^(45)^ | Y | Y | N | Y | Y | Y | N | Y | Y | Y | Y | Y | Y | Y | Y | Y | 14 | 0 | 2 | Low |
| 47 | Yunan He, 2023 ^(15)^ | Y | Y | PY | Y | Y | N | Y | Y | N | Y | Y | Y | Y | Y | Y | Y | 12 | 1 | 3 | Low |

Y=Yes, PY=Partial Yes, N=No, N/A=Not applicable, *critical items.

1. Did the research questions and inclusion criteria for the review include the components of PICO?
2. Did the report of the review contain an explicit statement that the review methods were established prior to the conduct of the review and did the report justify any significant deviations from the protocol?
3. Did the review authors explain their selection of the study designs for inclusion in the review?
4. Did the review authors use a comprehensive literature search strategy?
5. Did the review authors perform study selection in duplicate?
6. Did the review authors perform data extraction in duplicate?
7. Did the review authors provide a list of excluded studies and justify the exclusions?
8. Did the review authors describe the included studies in adequate detail?
9. Did the review authors use a satisfactory technique for assessing the risk of bias (RoB) in individual studies that were included in the review?
10. Did the review authors report on the sources of funding for the studies included in the review?
11. If meta-analysis was performed did the review authors use appropriate methods for statistical combination of results?
12. If meta-analysis was performed, did the review authors assess the potential impact of RoB in individual studies on the results of the meta-analysis or other evidence synthesis?
13. Did the review authors account for RoB in individual studies when interpreting/ discussing the results of the review?
14. Did the review authors provide a satisfactory explanation for, and discussion of, any heterogeneity observed in the results of the review?
15. If they performed quantitative synthesis did the review authors carry out an adequate investigation of publication bias (small study bias) and discuss its likely impact on the results of the review?
16. Did the review authors report any potential sources of conflict of interest, including any funding they received for conducting the review?

**Appendix S1**

Search terms for each database

- Medline

"embryo implantation"[MeSH Terms] OR ("embryo"[All Fields] AND "implantation"[All Fields]) OR "embryo implantation"[All Fields] Filters: Systematic Review

- Scopus

( TITLE-ABS-KEY ( implantation AND failure ) OR TITLE-ABS-KEY ( repeated AND implantation AND failure ) OR TITLE-ABS-KEY ( recurrent AND implantation AND failure ) OR TITLE-ABS-KEY ( rif ) AND TITLE-ABS-KEY ( systematic AND review ) OR TITLE-ABS-KEY ( meta-analysis ) ) AND ( LIMIT-TO ( DOCTYPE , "re" ) )

- Cochrane Library

recurrent implantation failure in Title Abstract Keyword OR RIF in Title Abstract Keyword OR implantation failure in Title Abstract Keyword AND systematic review in Title Abstract Keyword - (Word variations have been searched)
